# Supplementary material for: Pharmacological Properties of Polyphenols: Bioavailability, Mechanisms of Action, and Biological Effects in In Vitro Studies, Animal Models, and Humans
Source: Biomedicines. 2021 Aug 23;9(8):1074. doi: 10.3390/biomedicines9081074 (PMC8392236; doi:10.3390/biomedicines9081074)
Supplement: Supplementary file 1 [file biomedicines-09-01074-s001.zip › biomedicines-1321366-supplementary.pdf]

Supplementary material

Methodology

The literature review was performed by conducting bibliographic searches using the database PubMed.

The electronic search used the following keywords

- (I) (polyphenol[Title]) AND (bioavailability[Title/abstract])
- (II) (pharmacological[Title/abstract]) AND (polyphenol[Title]) AND (cardiovascular disease[Title]);
- (III) (II) (pharmacological[Title/abstract]) AND (polyphenol[Title]) AND (diabetes[Title]);
- (IV) (III) (pharmacological[Title/abstract]) AND (polyphenol[Title]) AND (obesity[Title]);
- (V) (IV) (pharmacological[Title/abstract]) AND (polyphenol[Title]) AND (digestive disease[Title]);
- (VI) (pharmacological[Title/abstract]) AND (polyphenol[Title]) AND (neurodegenerative disease[Title]);
- (VII) (pharmacological[Title/abstract]) AND (polyphenol[Title]) AND (cancer[Title]);

Inclusion criteria was set to studies published in the last 5 years.

Following the outlined searches, articles were chosen based on their relevance to the objective of this review. The articles providing information in clear relation to polyphenols and their pharmacological effects, with clear indication of their action mechanisms, were included. A total of 127 articles were included in this review.
